# A global assessment of the male predominance in esophageal adenocarcinoma

**DOI:** 10.18632/oncotarget.9113

**Published:** 2016-04-29

**Authors:** Shao-Hua Xie, Jesper Lagergren

**Affiliations:** ^1^ Upper Gastrointestinal Surgery, Department of Molecular Medicine and Surgery, Karolinska Institutet, Karolinska University Hospital, Stockholm, Sweden; ^2^ Section of Gastrointestinal Cancer, Division of Cancer Studies, King's College London, London, United Kingdom

**Keywords:** esophageal adenocarcinoma, sex difference, male predominance, incidence, epidemiology

## Abstract

**Background:**

Esophageal adenocarcinoma (EAC) is characterized by a male predominance. However, variations in the sex difference across populations and over time have not previously been thoroughly investigated.

**Results:**

The male-to-female ratio in EAC incidence varied greatly across continents, ranging from 1.03 in Africa to 7.64 in Northern America during 2003– 2007. The ratio was high in Europe (6.04) and Oceania (6.24), and lower in Asia (4.37) and Latin America and the Caribbean (3.94). The sex ratio remained relatively stable over time in most populations. In absolute terms, the sex difference in EAC incidence increased over time in populations of higher incidence, while it remained stable or slightly decreased in low-incidence populations.

**Materials and Methods:**

We used data from the Cancer Incidence in Five Continents series to compute sex-specific age-standardized rates of EAC by population. The sex difference in incidence was evaluated on both absolute and relative scales, measured by the absolute difference and ratio between sexes, respectively.

**Conclusions:**

This first global assessment of the sex ratio in EAC shows that the male predominance is particularly strong in developed countries. The underlying reasons remain to be identified, but the emerging EAC burden in men merits consideration for targeted prevention and early detection.

## INTRODUCTION

Esophageal adenocarcinoma (EAC) is one of the two main histological types of esophageal cancer. It carries a poor prognosis with an overall 5-year survival lower than 15% [1, 2], and the incidence has been rapidly increasing during the past four decades in Western societies, particularly in white males [2, 3]. In 2012, there were 52,000 new patients diagnosed with EAC globally, and the highest incidence globally is noted in the United Kingdom (UK) [4].

EAC continues to fascinate researchers and others with its striking and enigmatic male predominance, with a male-to-female ratio in incidence of 4–5:1 on average [2–6]. The male predominance is not readily explained by sex differences in the exposure to the established risk factors for EAC, i.e., obesity, gastroesophageal reflux disease, *Helicobacter pylori* (*H. pylori*) infection (inverse association) or tobacco smoking [2, 5, 7]. Instead, it has been hypothesized that sex hormones and reproductive factors might play a role in the development of EAC or its precancerous lesion Barrett's esophagus, although the existing evidence is far from conclusive [2, 5, 8].

Variations in the male predominance of EAC across populations and over time have, to the best of our knowledge, not previously been thoroughly investigated. A global assessment of these variations might have important etiological implications. Great variations in the sex difference in EAC incidence across populations with the same ethnicity or over time in the same population are expected to be predominantly attributable to environmental risk factors, while a stable sex ratio within a population indicates a key role of the sex itself or intrinsic exposures related to sex, including hormonal or genetic factors. Therefore, we performed a global evaluation of the patterns of the sex difference in the incidence of EAC.

## RESULTS

### Continents

**Figure 1:**
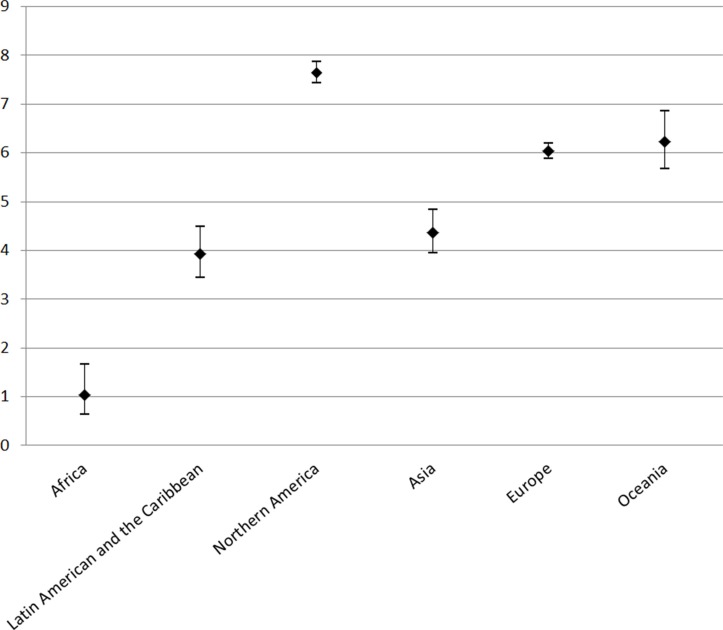
Male-to-female ratios in age-standardized incidence rates of esophageal adenocarcinoma and their 95% confidence intervals by continent

As shown in Figure 1, the male-to-female ratio in EAC incidence varied greatly across continents during the period 2003–2007, ranging from the lowest 1.03 (95% confidence interval [CI]: 0.64, 1.64) in Africa to 7.64 (95% CI: 7.43, 7.86) in Northern America. In other continents, the ratio was high in Europe (6.04, 95% CI: 5.88, 6.20) and Oceania (6.24, 95% CI: 5.68, 6.85), but lower in Asia 4.37 (95% CI: 3.95, 4.84) and Latin America and the Caribbean 3.94 (95% CI: 3.45, 4.50).

### Countries

On a country level in 2003–2007, the lowest sex ratio in EAC was observed in Iran (0.98, 95% CI: 0.53, 1.79), while the highest was found in Lithuania (9.54, 95% CI: 5.45, 16.68). Data from countries with higher overall incidence rates of EAC (both sexes) provided more precise estimates as indicated by narrower CIs (Supplementary Table 1). The male-to-female ratios in EAC incidence in the Western countries the UK, the US, Canada, Australia, and the Netherlands were 4.91 (95% CI: 4.74, 5.09), 7.73 (95% CI: 7.51, 7.97), 6.87 (95% CI: 6.25, 7.55), 6.25 (95% CI: 5.63, 6.93), and 5.70 (95% CI: 5.28, 6.15), respectively. In Asian countries, high sex ratios were observed in Japan (7.17, 95% CI: 5.5, 9.35) and the Republic of Korea (6.82, 95% CI: 5.23, 8.91), while these ratios were lower in other Asian countries. The sex ratios were higher in countries with higher incidence rates (Supplementary Figure 1). The ethnicity-specific sex ratios in the US were higher in Whites than in Asian, Pacific Islanders and Blacks (Table 1). In terms of absolute risk difference, the sex difference in EAC incidence was highest in the UK (5.43 per 100 000 person-years, 95% CI: 5.31, 5.55), followed by the Netherlands (5.32, 95% CI: 5.10, 5.54), Ireland (4.22, 95% CI: 3.77, 4.67), and the US (3.49, 95% CI: 3.44, 3.54). More detailed results for all countries and regions included in this study are provided in the Supplementary Materials (Supplementary Table 1).

**Table 1:** Sex difference in the incidence of esophageal adenocarcinoma by ethnicity in the United States of America (NPCR 42 states), 2003–2007

Ethnicities	No. of cases		ASIR (95% CI)^a^		RD (95% CI)^a^	RR (95% CI)
	Males	Females	Males	Females		
White	31551	5209	4.45 (4.40, 4.50)	0.57 (0.55, 0.58)	3.88 (3.83, 3.93)	7.85 (7.61, 8.10)
Asian and Pacific Islander	145	38	0.53 (0.45, 0.63)	0.11 (0.08, 0.15)	0.43 (0.33, 0.52)	5.02 (3.50, 7.19)
Black	695	249	0.94 (0.87, 1.02)	0.24 (0.21, 0.27)	0.71 (0.63, 0.78)	3.97 (3.43, 4.60)
American Indian	120	23	2.02 (1.67, 2.43)	0.35 (0.22, 0.52)	1.67 (1.28, 2.07)	5.79 (3.69, 9.08)
All	32741	5565	4.01 (3.96, 4.05)	0.52 (0.50, 0.53)	3.49 (3.44, 3.54)	7.73 (7.51, 7.97)

ASIR: age-standardized incidence rate using the World Health Organization (WHO) World Standard Population 2000 as the reference (1/100 000 person-years); CI: confidence interval; NPCR: National Program of Cancer Registries; RD: risk difference; RR: relative risk measured by the male-to-female ratio in the age-standardized incidence rate.

a In 1/100 000 person-years.

### Calendar time

**Figure 2:**
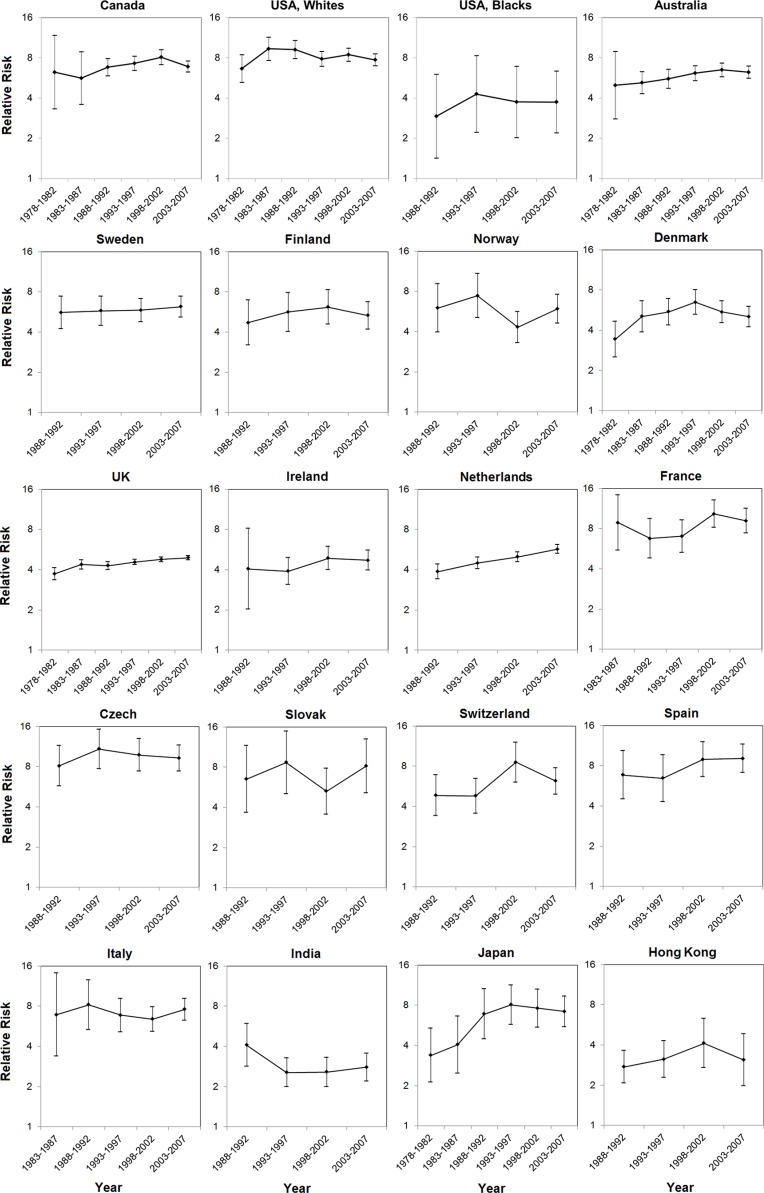
Male-to-female ratios in age-standardized incidence rates of esophageal adenocarcinoma and their 95% confidence intervals by calendar period in selected populations

**Figure 3:**
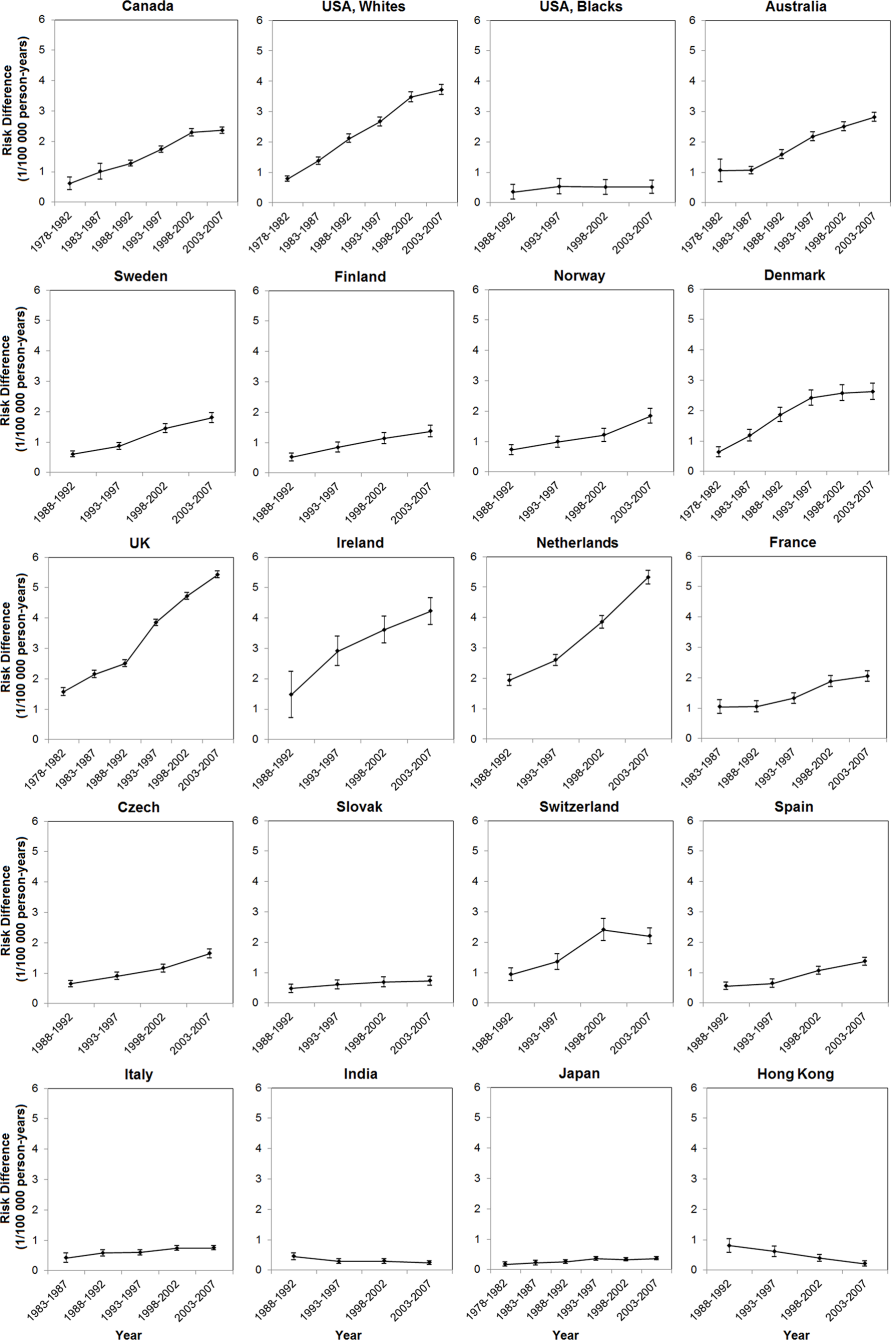
Risk differences in age-standardized incidence rates of esophageal adenocarcinoma between the sexes (1/100 000 person-years) and their 95% confidence intervals by calendar period in selected populations

The male-to-female ratio in EAC incidence has remained relatively stable since the 1970 s or 1980 s in most populations, whereas a seemingly steady increase was noted in the UK (from 3.73 [95% CI: 3.36, 4.14] during 1978–1982 to 4.91 [95% CI: 4.74, 5.09] during 2003– 2007) and the Netherlands (from 3.87 [95% CI: 3.41, 4.39] during 1988–1992 to 5.70 [95% CI: 5.28, 6.15] during 2003–2007). The sex ratios in Canada, Australia, Denmark, and Japan increased from the period 1978–1982 until the mid-to-late 1990 s, but remained stable or showed a slight decrease thereafter (Figure 2, Supplementary Table 2). In absolute terms, the sex difference in EAC incidence steadily increased over time in populations of higher incidence, but remained stable or slightly decreased in low-incidence populations (Figure 3, Supplementary Table 2).

## DISCUSSION

The present study identified a worldwide male predominance in EAC, which was stronger in developed countries. The sex ratio in EAC incidence remained relatively stable over time in most populations, except for an increase in a few countries, particularly in the UK and the Netherlands. The *absolute* difference in EAC incidence between the sexes steadily increased over time in the Western populations, indicating an emerging global burden of EAC in men.

The present study found that the global male predominance in EAC is more pronounced in Western populations, namely, Northern America, Europe, and Oceania. In Asia, the male-to-female ratios in EAC incidence were generally lower, although they were high in Japan and the Republic of Korea. These latter countries are more developed (“semi-Western”) countries compared with other Asian countries. The increasing trend of the male predominance from the late 1970 s to the mid-1990 s in Japan is in line with the post-war economic growth coupled with modernization in Japanese society. The findings of extreme male predominance in EAC in the developed world may be attributable to risk factors related to Western lifestyle. A recent analysis suggested higher male to female ratio in EAC incidence in populations with higher prevalence of obesity [9], which may partially explain the more pronounced male predominance in these more developed countries. However, the sex ratios in EAC incidence did not substantially change over time in most populations, suggesting a key role of intrinsic exposures (e.g., genetic factors or sex hormones), or some environmental exposures with stable differences in prevalence between the sexes. Furthermore, the sex ratios in EAC incidence in immigrants in the US were similar to those in their regions of origin, which indicates that genetic or ethnicity-specific lifestyle factors might be involved in explaining the male predominance of EAC. The slight but notable increased male-to-female ratio in EAC incidence in Canada, Japan, and possibly in the UK may, to some extent, be explained by more rapidly increased prevalence of obesity in men in these countries since the 1980s. However, the increased sex ratio in EAC incidence over time in the Netherlands, Australia, and Denmark does not match the relatively stable sex ratios in the prevalence of obesity in these countries (Supplementary Figure 2) [10].

Despite the generally stable sex ratios over time, the *absolute* difference between the sexes in EAC incidence has increased in all Western countries included in this study. This emerging burden of EAC in men may have implications for public health decision-makers in designing and implementing targeted prevention and measures of early detection of this cancer. The decreasing absolute sex difference in EAC incidence in Hong Kong parallels the decrease in prevalence of tobacco smoking in the Hong Kong population, which is more pronounced in women [11].

The male predominance in EAC is not readily explained by the main established risk factors for EAC, i.e., obesity, reflux, and *H. pylori* infection (inverse), since the prevalences of these exposures are similar between the sexes and existing evidence does not support any stronger association between these factors and EAC in men than in women [2, 5, 12–14]. Abdominal obesity, as the typical male fat distribution and a risk factor for EAC [15], has been hypothesized to contribute to the male predominance in EAC. However, this hypothesis was not supported by a stratified analysis showing no increased male predominance among overweight EAC patients compared with lean [16]. Reflux disease seems to be more severe in men than in women [17], which may partially explain the male predominance in EAC as erosive reflux disease, which is a stronger risk factor for EAC than non-erosive reflux [18]. Accordingly, reflux seems to be more prevalent in men than in women and the prevalence of this condition has increased in recent decades in Japan [19, 20], which is in line with the increased male-to-female ratio in EAC incidence observed in this study. Tobacco smoking is more prevalent in Western women than in Asian women, which does not match the more distinct male predominance in EAC in Western countries. Moreover, there is no stronger association between tobacco smoking and EAC risk in men than in women, and the male predominance in EAC is similar in smokers and non-smokers [7]. Thus, the male predominance in EAC is unlikely to be explained by tobacco smoking, particularly not for the Western populations. Some yet unidentified risk factors associated with the Western lifestyle which are more prevalent or harmful in men may have contributed to the male predominance in EAC, and merit further investigation.

A 16-year delayed development of EAC in women compared to men has been noted, suggesting a protective role of endogenous estrogen in the development of EAC [21]. The existing evidence regarding a potential association between estrogen exposure and risk of EAC or its precancerous lesion, Barrett's esophagus, remains inconclusive, however [2, 5, 8]. Based on the hypothesized protective role of estrogenic exposures, the increased male-to-female ratios over time observed in several populations included in this study may be partially explained by the increasing use of oral contraceptives and hormonal replacement therapy [22], which may have resulted in a decrease of EAC risk in women in these countries. Yet, the role of sex hormonal factors in EAC development remains to be established in valid and large population-based studies.

Another noteworthy finding of this study is that the incidence rates of EAC were virtually the same for both sexes in Iran. Interestingly, more similar incidence rates between the sexes than in other countries have also been observed for other types of male-predominant cancers, e.g., hepatocellular carcinoma, in Iran compared with other parts of world [23]. Such findings suggest distinct etiological profiles of gastrointestinal malignancies in the Iranian population, which may include some exposures more equally distributed between the sexes, e.g. shared dietary factors.

EAC carries a poor prognosis with an overall 5-year survival lower than 15% in Western populations, and tumor stage at diagnosis is by far the strongest prognostic factor [1, 2]. Thus, detection at an early stage would have the potential to greatly reduce the mortality of EAC. Upper endoscopy has been increasingly used for the detection of EAC or its precursor lesion, Barrett's esophagus, particularly in patients with reflux symptoms. However, an unselective endoscopic surveillance, even among reflux patients, seems infeasible considering the low absolute risk of EAC in the population, the considerable costs, the invasiveness of the procedure, as well as its error prone nature, due to sampling bias and subjective diagnosis. Instead, targeting a limited group of individuals at high risk of EAC would be necessary, and the strong male predominance in EAC needs to be considered when selecting such high-risk groups. The Clinical Guidelines Committee of the American College of Physicians proposed upper endoscopy in men aged > 50 years with long-lasting reflux symptoms and other risk factors [24]. However, the benefits and risks of such a practice remain to be carefully weighted before it is adopted in other populations given the differential epidemiological features, including the variations in the sex difference in incidence across populations.

This study is the first to thoroughly investigate the variations in the male predominance of EAC across populations and over time globally. Most previous studies have measured the sex difference in EAC incidence in relative terms, which are less likely to be affected by changes in absolute rates and can facilitate comparisons between populations. However, the absolute disparity between the sexes could not have been reflected. In this study, we measured the sex difference in EAC incidence on both absolute and relative scales, which have provided fundamentally different types of information. Moreover, the data from the employed cancer registers were of good quality [25–29]. A limitation is the risk of misclassification of histological typing, which may have resulted in some underestimation of EAC incidence in some registers, particularly in earlier periods. Moreover, the classification standards might vary between registers. However, it is unlikely that any misclassification or underreporting would have been differential between the sexes, and thus, should not influence the sex ratios in EAC incidence to a great extent. The possible histological misclassification might have greater impact on the estimated ratios in populations with high proportions of squamous cell carcinoma given that even a small proportion of misclassification from squamous cell carcinoma to adenocarcinoma might have led to substantial artificial changes in the estimated incidence rates in these populations. Such possible histological misclassification may be an alternative explanation for the lower male to female ratios in EAC incidence in populations with low incidence of EAC but higher incidence of squamous cell carcinoma. In addition, it cannot be ruled out that the absolute risk differences between the sexes may have been underestimated if inaccurate histological classification existed.

In conclusion, this first global assessment of the sex ratio in EAC identified a clearly stronger male predominance in Western countries. The observed male predominance, in terms of both geographical variations and changes over time, might be due to a combination of environmental, hormonal and genetic factors, but more research is needed. The increasing differences in absolute number of cases in men compared to women indicates an emerging global burden of EAC in men, which needs to be taken into consideration when designing and implementing targeted prevention and measures of early tumor detection.

## MATERIALS AND METHODS

### Data sources

We extracted EAC incidence and population data from the *Cancer Incidence in Five Continents* (CI5) series, which are monographs published by the International Agency for Research on Cancer (IARC) containing information on cancer incidence from all over the world where good quality data are available [25, 27–29]. The volumes VII to X, containing detailed data with morphological coding, were used since it is otherwise not possible to distinguish EAC from the other main histological type of esophageal cancer, i.e., squamous cell carcinoma. We further extracted data from the 1970 s or early 1980 s from the *CI5plus* database, which contains updated annual incidence rates for selected populations up to the year 2007 [26]. Registers without any EAC patients in each volume were excluded. We pooled the numbers of cases and population sizes from multiple regional registers within a country if there was no nationwide data available. Countries with fewer than ten EAC patients for each sex in each volume were excluded due to statistical instability from including extremely low incidence rates, or possible incomplete histological coding.

### Statistical analyses

All statistical analyses were performed using the statistical software SAS 9.4 (SAS Institute, Cary, NC). We first calculated the sex-specific crude and age-standardized incidence rates (ASIRs) by country and region for each CI5 volume in five-year calendar periods. The ASIRs were calculated using the direct method with the World Health Organization (WHO) World Standard Population 2000 as the reference [30]. The 95% CIs of crude rates were calculated under the assumption of Poisson distribution, while CIs for ASIRs were computed based on the gamma distribution as it assumes that the standardized rate is a weighted sum of independent Poisson random variables [31, 32].

Indicators of sex differences in EAC incidence were calculated on both absolute and relative scales, namely risk difference and relative risk, respectively. Risk differences were calculated by subtracting the ASIR in males from that in females, while relative risks were measured as the male-to-female ratio in ASIR. The 95% CIs of risk differences and relative risks were estimated based on the assumptions of normal and log-normal distributions, respectively [32]. In the global comparisons, we present results from the most recent CI5 volume (number X) from 2003 to 2007, which contains the most complete coverage and highest quality of registers, including good information on EAC diagnosis. We further pooled the estimates for six major regions in the world (Africa, Latin America and the Caribbean, Northern America, Asia, Europe, and Oceania) based on the geographical definition of the United Nations' World Population Prospects [33]. In addition, we estimated the sex difference in EAC incidence by ethnic groups in the United States (US). Time trends in the sex difference of EAC incidence were evaluated in 20 selected populations with available data in each of the four CI5 volumes.

## SUPPLEMENTARY MATERIALS FIGURES AND TABLES


