# Supplementary material for: A global assessment of the male predominance in esophageal adenocarcinoma
Source: Oncotarget. 2016 Apr 29;7(25):38876–83. doi: 10.18632/oncotarget.9113 (PMC5122437; doi:10.18632/oncotarget.9113)
Supplement: Supplementary file 1 [file oncotarget-07-38876-s001.pdf]

A global assessment of the male predominance in esophageal adenocarcinoma

Supplementary Materials

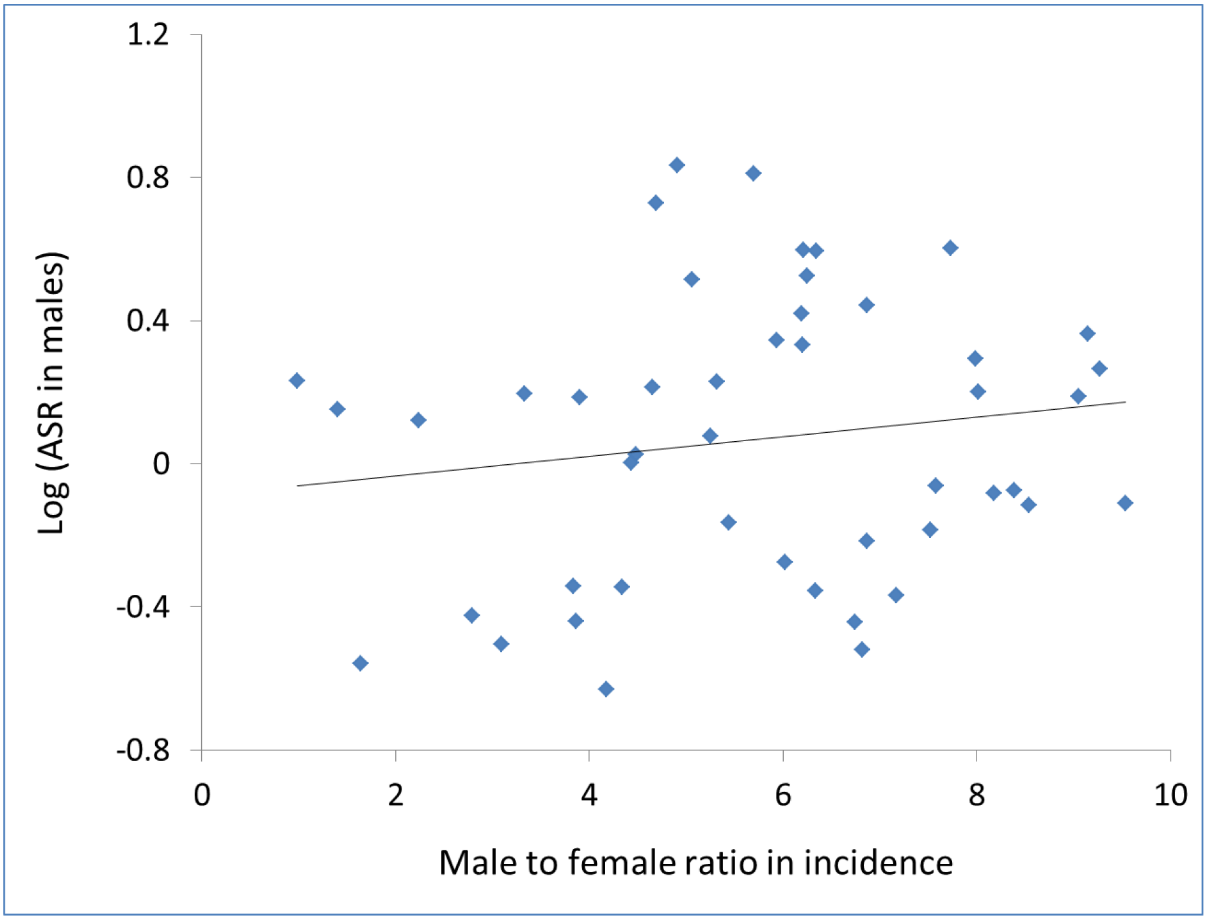

Supplementary Figure S1: Scatter plot of the male-to-female ratio in the age-standardized incidence rates of esophageal adenocarcinoma against the logarithm of age-standardized incidence rates in males in selected countries during the period 2003–2007.

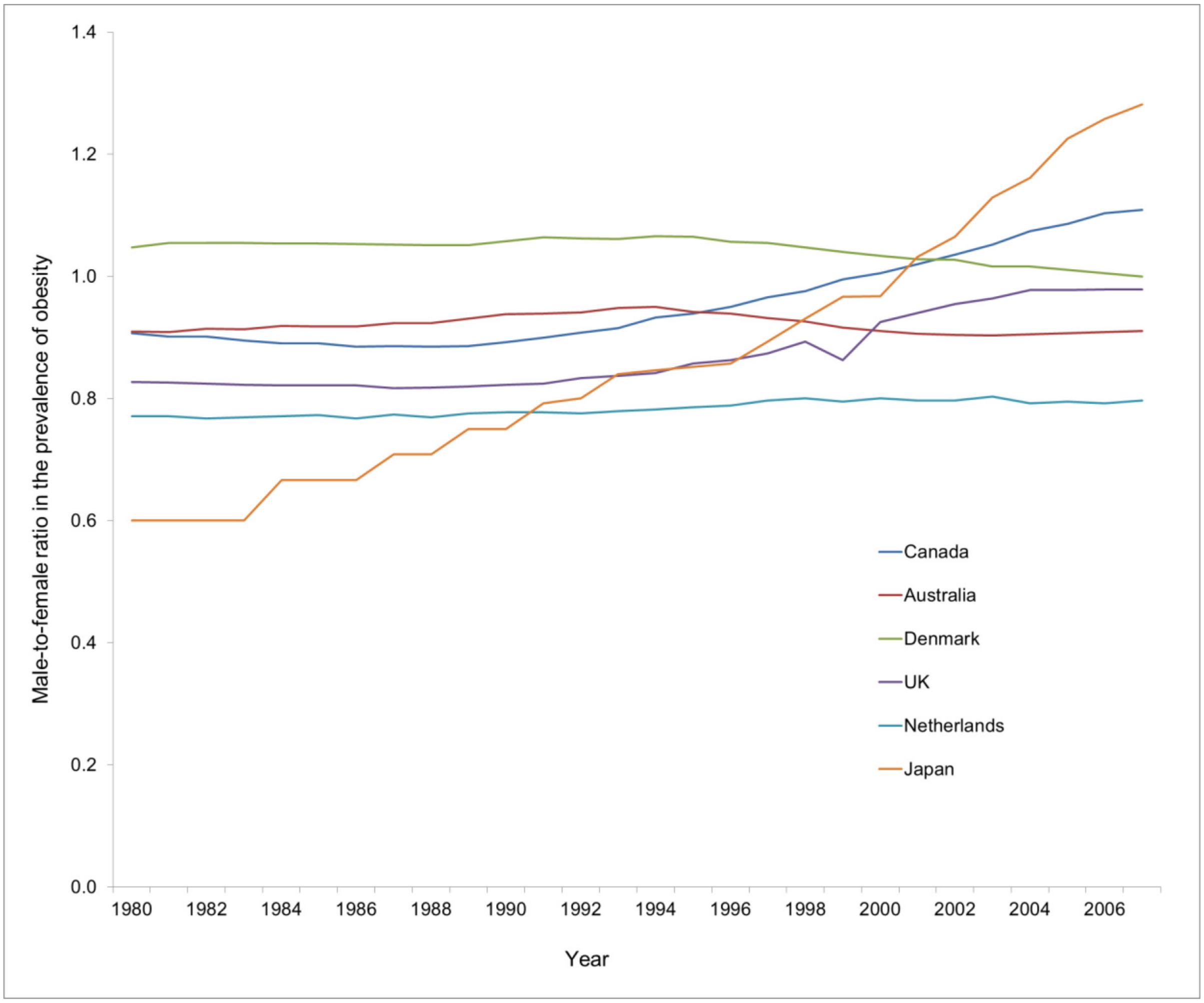

Supplementary Figure S2: The male-to-female ratios in the age-standardized prevalence of obesity (body mass index ≥ 30) among adults in selected countries during the period 1980–2007. Data source: Institute for Health Metrics and Evaluation (IHME). Overweight and Obesity Viz. Seattle, WA: IHME, University of Washington, 2014. Available from http://vizhub.healthdata.org/obesity. (Accessed on16 October 2015).

Supplemental Table S1: Sex difference in the incidence of esophageal adenocarcinoma by country/ region in cancer incidence in five continents volume X

Countries/ regions	No. of cases		Crude incidence rate (95% CI)[a]		ASIR (95% CI)[a]		RD (95% CI)[a]	RR (95% CI)
	Males	Females	Males	Females	Males	Females		
South Africa	19	29	0.78 (0.47, 1.22)	0.98 (0.66, 1.41)	1.42 (0.85, 2.22)	1.01 (0.66, 1.49)	0.41 (–0.35, 1.16)	1.40 (0.77, 2.55)
Egypt	16	10	0.16 (0.09, 0.26)	0.10 (0.05, 0.19)	0.28 (0.15, 0.46)	0.17 (0.08, 0.31)	0.11 (–0.07, 0.29)	1.64 (0.71, 3.78)
**Africa**	35	39	0.28 (0.20, 0.39)	0.31 (0.22, 0.42)	0.50 (0.34, 0.69)	0.48 (0.34, 0.66)	0.02 (–0.22, 0.25)	1.03 (0.64, 1.66)
Argentina	119	36	1.58 (1.31, 1.89)	0.45 (0.32, 0.62)	1.63 (1.35, 1.95)	0.35 (0.24, 0.49)	0.28 (–0.04, 0.60)	4.65 (3.18, 6.81)
Brazil	436	165	1.15 (1.04, 1.26)	0.39 (0.34, 0.46)	1.53 (1.39, 1.69)	0.39 (0.33, 0.46)	1.14 (0.98, 1.31)	3.90 (3.22, 4.72)
Chile	24	13	1.03 (0.66, 1.53)	0.58 (0.31, 1.00)	1.32 (0.84, 1.96)	0.59 (0.31, 1.01)	0.73 (0.11, 1.35)	2.24 (1.14, 4.41)
Colombia	73	23	0.80 (0.63, 1.01)	0.23 (0.15, 0.34)	1.06 (0.83, 1.33)	0.24 (0.15, 0.36)	0.82 (0.56, 1.09)	4.48 (2.77, 7.23)
USA, Puerto Rico	145	39	1.54 (1.30, 1.82)	0.38 (0.27, 0.52)	1.19 (1.00, 1.41)	0.23 (0.16, 0.32)	0.97 (0.75, 1.18)	5.25 (3.64, 7.57)
Uruguay	93	42	1.94 (1.56, 2.37)	0.82 (0.59, 1.10)	1.56 (1.26, 1.92)	0.47 (0.33, 0.66)	1.10 (0.74, 1.45)	3.33 (2.27, 4.89)
**Latin America and the Caribbean**	890	318	1.25 (1.17, 1.34)	0.41 (0.37, 0.46)	1.42 (1.32, 1.52)	0.36 (0.32, 0.40)	1.06 (0.95, 1.16)	3.94 (3.45, 4.50)
Canada	3027	560	3.79 (3.66, 3.93)	0.69 (0.63, 0.75)	2.76 (2.67, 2.87)	0.40 (0.37, 0.44)	2.36 (2.26, 2.47)	6.87 (6.25, 7.55)
USA,NPCR (42 states)	32741	5565	5.04 (4.99, 5.10)	0.83 (0.81, 0.85)	4.01 (3.96, 4.05)	0.52 (0.50, 0.53)	3.49 (3.44, 3.54)	7.73 (7.51, 7.97)
**Northern America**	35768	6125	4.91 (4.85, 4.96)	0.82 (0.80, 0.84)	3.86 (3.82, 3.90)	0.50 (0.49, 0.52)	3.36 (3.31, 3.40)	7.64 (7.43, 7.86)
Mainland China	301	87	0.63 (0.56, 0.71)	0.19 (0.15, 0.23)	0.46 (0.40, 0.51)	0.12 (0.09, 0.15)	0.34 (0.28, 0.39)	3.83 (3.01, 4.88)
Hong Kong	71	30	0.43 (0.34, 0.55)	0.17 (0.11, 0.24)	0.31 (0.24, 0.40)	0.10 (0.07, 0.15)	0.21 (0.13, 0.29)	3.10 (1.98, 4.84)
India	265	96	0.23 (0.20, 0.25)	0.10 (0.08, 0.12)	0.38 (0.33, 0.43)	0.14 (0.11, 0.17)	0.24 (0.19, 0.30)	2.79 (2.19, 3.55)
Iran	25	21	1.01 (0.65, 1.49)	0.86 (0.53, 1.32)	1.70 (1.10, 2.52)	1.74 (1.04, 2.68)	–0.03 (–1.07, 1.00)	0.98 (0.54, 1.79)
Israel	169	53	1.03 (0.88, 1.20)	0.32 (0.24, 0.41)	1.00 (0.86, 1.17)	0.23 (0.17, 0.30)	0.78 (0.61, 0.94)	4.44 (3.22, 6.10)
Japan	373	79	0.79 (0.71, 0.87)	0.16 (0.12, 0.20)	0.43 (0.38, 0.48)	0.06 (0.05, 0.08)	0.37 (0.32, 0.42)	7.17 (5.50, 9.35)
Republic of Korea	312	67	0.26 (0.23, 0.29)	0.06 (0.04, 0.07)	0.30 (0.27, 0.34)	0.04 (0.03, 0.06)	0.26 (0.22, 0.29)	6.82 (5.23, 8.91)
Philippines	59	18	0.19 (0.15, 0.25)	0.06 (0.03, 0.09)	0.45 (0.33, 0.60)	0.10 (0.06, 0.17)	0.35 (0.21, 0.49)	4.34 (2.49, 7.57)
Thailand	53	15	0.21 (0.16, 0.27)	0.05 (0.03, 0.09)	0.23 (0.17, 0.31)	0.06 (0.03, 0.09)	0.18 (0.11, 0.25)	4.18 (2.34, 7.46)
Turkey	70	13	0.47 (0.36, 0.59)	0.09 (0.05, 0.15)	0.53 (0.41, 0.67)	0.09 (0.05, 0.15)	0.44 (0.31, 0.58)	6.02 (3.32, 10.91)
**Asia**	1698	479	0.39 (0.37, 0.40)	0.11 (0.10, 0.12)	0.41 (0.39, 0.42)	0.09 (0.08, 0.10)	0.31 (0.29, 0.33)	4.37 (3.95, 4.84)
Austria	468	81	2.34 (2.14, 2.57)	0.38 (0.30, 0.48)	1.59 (1.44, 1.74)	0.20 (0.15, 0.25)	1.39 (1.24, 1.54)	8.02 (6.22, 10.33)
Belgium	1259	293	6.13 (5.80, 6.48)	1.37 (1.22, 1.53)	3.92 (3.70, 4.15)	0.62 (0.54, 0.70)	3.30 (3.07, 3.54)	6.35 (5.54, 7.28)
Bulgaria	127	30	0.68 (0.56, 0.80)	0.15 (0.10, 0.21)	0.44 (0.37, 0.53)	0.07 (0.05, 0.11)	0.37 (0.29, 0.45)	6.34 (4.19, 9.58)
Belarus	201	45	0.88 (0.76, 1.01)	0.17 (0.13, 0.23)	0.77 (0.66, 0.88)	0.09 (0.06, 0.12)	0.68 (0.56, 0.79)	8.54 (6.07, 12.00)
Croatia	102	21	0.95 (0.78, 1.16)	0.18 (0.11, 0.28)	0.65 (0.53, 0.80)	0.09 (0.05, 0.14)	0.57 (0.43, 0.70)	7.53 (4.53, 12.50)
Czech Republic	649	98	2.60 (2.40, 2.80)	0.37 (0.30, 0.46)	1.84 (1.70, 2.00)	0.20 (0.16, 0.25)	1.65 (1.50, 1.80)	9.27 (7.42, 11.58)
Denmark	687	175	5.12 (4.75, 5.52)	1.28 (1.10, 1.48)	3.27 (3.03, 3.53)	0.65 (0.55, 0.76)	2.62 (2.36, 2.89)	5.06 (4.25, 6.03)
Finland	335	96	2.61 (2.34, 2.90)	0.72 (0.58, 0.87)	1.69 (1.51, 1.89)	0.32 (0.25, 0.40)	1.37 (1.18, 1.57)	5.32 (4.20, 6.75)
France	721	117	3.54 (3.28, 3.81)	0.55 (0.45, 0.65)	2.30 (2.13, 2.48)	0.25 (0.20, 0.31)	2.05 (1.87, 2.23)	9.15 (7.41, 11.3)
Germany	1722	308	3.30 (3.14, 3.46)	0.56 (0.50, 0.63)	1.96 (1.87, 2.06)	0.25 (0.22, 0.28)	1.72 (1.62, 1.82)	7.99 (7.00, 9.12)

Ireland	647	176	6.26 (5.78, 6.76)	1.69 (1.45, 1.96)	5.36 (4.96, 5.79)	1.14 (0.97, 1.33)	4.22 (3.77, 4.67)	4.70 (3.95, 5.58)
Italy	790	157	1.56 (1.45, 1.67)	0.29 (0.25, 0.34)	0.87 (0.80, 0.93)	0.11 (0.10, 0.14)	0.75 (0.69, 0.82)	7.58 (6.28, 9.15)
Lithuania	78	16	0.98 (0.77, 1.22)	0.18 (0.10, 0.29)	0.78 (0.61, 0.97)	0.08 (0.05, 0.15)	0.69 (0.52, 0.87)	9.54 (5.45, 16.68)
The Netherlands	3764	894	9.33 (9.03, 9.63)	2.17 (2.03, 2.32)	6.45 (6.25, 6.67)	1.13 (1.06, 1.21)	5.32 (5.10, 5.54)	5.70 (5.28, 6.15)
Norway	374	92	3.26 (2.93, 3.60)	0.79 (0.64, 0.97)	2.21 (1.99, 2.45)	0.37 (0.29, 0.47)	1.84 (1.59, 2.08)	5.93 (4.63, 7.60)
Poland	77	16	0.46 (0.36, 0.58)	0.09 (0.05, 0.15)	0.36 (0.28, 0.45)	0.05 (0.03, 0.09)	0.31 (0.22, 0.39)	6.74 (3.87, 11.76)
Russian Federation	88	36	0.86 (0.69, 1.05)	0.28 (0.20, 0.39)	0.69 (0.55, 0.85)	0.13 (0.09, 0.19)	0.56 (0.41, 0.71)	5.45 (3.64, 8.15)
Slovakia	124	22	0.95 (0.79, 1.13)	0.16 (0.10, 0.24)	0.83 (0.69, 0.99)	0.10 (0.06, 0.16)	0.73 (0.57, 0.88)	8.18 (5.14, 13.01)
Slovenia	58	10	1.18 (0.90, 1.53)	0.20 (0.09, 0.36)	0.84 (0.64, 1.10)	0.10 (0.05, 0.21)	0.74 (0.51, 0.97)	8.39 (4.11, 17.1)
Spain	622	88	2.34 (2.16, 2.53)	0.33 (0.26, 0.40)	1.54 (1.42, 1.67)	0.17 (0.13, 0.22)	1.37 (1.24, 1.50)	9.05 (7.09, 11.55)
Sweden	813	180	3.63 (3.38, 3.88)	0.79 (0.68, 0.91)	2.14 (2.00, 2.30)	0.35 (0.29, 0.41)	1.80 (1.64, 1.96)	6.20 (5.18, 7.42)
Switzerland	440	103	4.18 (3.80, 4.59)	0.93 (0.76, 1.13)	2.62 (2.38, 2.89)	0.42 (0.34, 0.53)	2.20 (1.94, 2.46)	6.20 (4.93, 7.78)
Ukraine	858	216	0.79 (0.74, 0.84)	0.17 (0.15, 0.19)	0.61 (0.57, 0.65)	0.09 (0.08, 0.10)	0.52 (0.48, 0.56)	6.86 (5.87, 8.03)
UK	15773	4536	10.69 (10.53, 10.86)	2.95 (2.87, 3.04)	6.82 (6.71, 6.93)	1.39 (1.34, 1.43)	5.43 (5.31, 5.55)	4.91 (4.74, 5.09)
Serbia	76	22	0.58 (0.45, 0.72)	0.16 (0.10, 0.24)	0.36 (0.28, 0.46)	0.09 (0.06, 0.15)	0.27 (0.18, 0.36)	3.86 (2.37, 6.29)
**Europe**	30853	7827	4.34 (4.29, 4.39)	1.03 (1.01, 1.05)	2.91 (2.87, 2.94)	0.48 (0.47, 0.49)	2.43 (2.39, 2.46)	6.04 (5.88, 6.20)
Australia	2303	470	4.62 (4.43, 4.81)	0.93 (0.85, 1.02)	3.35 (3.21, 3.49)	0.54 (0.49, 0.59)	2.81 (2.66, 2.96)	6.25 (5.63, 6.93)
New Zealand	522	108	5.16 (4.73, 5.63)	1.03 (0.85, 1.24)	3.95 (3.61, 4.30)	0.64 (0.52, 0.78)	3.31 (2.95, 3.67)	6.21 (5.00, 7.70)
**Oceania**	2825	578	4.71 (4.54, 4.89)	0.95 (0.87, 1.03)	3.44 (3.32, 3.57)	0.55 (0.51, 0.60)	2.89 (2.75, 3.03)	6.24 (5.68, 6.85)

ASIR: age-standardized incidence rate using the World Health Organization (WHO) World Standard Population 2000 as the reference; CI: confidence interval; NPCR: National Program of Cancer Registries; RD: risk difference; RR: relative risk measured by the male-to-female ratio in the age-standardized incidence rate.

[a]In 1/100,000 person-years.

Supplemental Table S2: Time trends of the sex difference in the incidence of esophageal adenocarcinoma in selected populations

Population	Period	RD (95% CI)[a]	RR (95% CI)
Canada	1978–1982	0.62 (0.41, 0.83)	6.24 (3.32, 11.74)
	1983–1987	1.01 (0.75, 1.27)	5.64 (3.58, 8.88)
	1988–1992	1.28 (1.18, 1.37)	6.80 (5.87, 7.87)
	1993–1997	1.74 (1.64, 1.84)	7.27 (6.43, 8.23)
	1998–2002	2.29 (2.17, 2.42)	8.09 (7.13, 9.18)
	2003–2007	2.36 (2.26, 2.47)	6.87 (6.25, 7.55)
USA, Whites[b]	1978–1982	0.78 (0.69, 0.88)	6.64 (5.23, 8.43)
	1983–1987	1.38 (1.26, 1.49)	9.34 (7.66, 11.38)
	1988–1992	2.11 (1.98, 2.25)	9.22 (7.88, 10.78)
	1993–1997	2.67 (2.51, 2.82)	7.84 (6.89, 8.91)
	1998–2002	3.47 (3.30, 3.64)	8.43 (7.53, 9.44)
	2003–2007	3.71 (3.55, 3.88)	7.72 (6.97, 8.54)
USA, Blacks[b]	1988–1992	0.35 (0.11, 0.59)	2.93 (1.42, 6.01)
	1993–1997	0.53 (0.28, 0.78)	4.28 (2.21, 8.28)
	1998–2002	0.51 (0.27, 0.75)	3.74 (2.03, 6.89)
	2003–2007	0.51 (0.29, 0.73)	3.73 (2.19, 6.36)
Australia	1978–1982	1.06 (0.68, 1.43)	4.98 (2.79, 8.90)
	1983–1987	1.07 (0.95, 1.19)	5.21 (4.31, 6.29)
	1988–1992	1.59 (1.44, 1.74)	5.57 (4.72, 6.57)
	1993–1997	2.18 (2.03, 2.32)	6.14 (5.40, 6.99)
	1998–2002	2.51 (2.36, 2.65)	6.50 (5.78, 7.31)
	2003–2007	2.81 (2.66, 2.96)	6.25 (5.63, 6.93)
Sweden	1988–1992	0.60 (0.50, 0.70)	5.61 (4.25, 7.41)
	1993–1997	0.87 (0.75, 0.98)	5.77 (4.49, 7.40)
	1998–2002	1.45 (1.30, 1.60)	5.83 (4.79, 7.09)
	2003–2007	1.80 (1.64, 1.96)	6.20 (5.18, 7.42)
Finland	1988–1992	0.52 (0.39, 0.65)	4.71 (3.19, 6.94)
	1993–1997	0.84 (0.68, 1.01)	5.65 (4.03, 7.91)
	1998–2002	1.13 (0.95, 1.31)	6.15 (4.59, 8.25)
	2003–2007	1.37 (1.18, 1.57)	5.32 (4.20, 6.75)
Norway	1988–1992	0.73 (0.57, 0.90)	6.01 (3.96, 9.12)
	1993–1997	0.99 (0.81, 1.17)	7.43 (5.07, 10.88)
	1998–2002	1.22 (1.00, 1.43)	4.32 (3.30, 5.66)
	2003–2007	1.84 (1.59, 2.08)	5.93 (4.63, 7.60)
Denmark	1978–1982	0.64 (0.48, 0.80)	3.42 (2.52, 4.65)
	1983–1987	1.18 (1.00, 1.38)	5.08 (3.88, 6.64)

	1988–1992	1.86 (1.64, 2.10)	5.49 (4.39, 6.88)
	1993–1997	2.42 (2.17, 2.68)	6.49 (5.26, 8.01)
	1998–2002	2.58 (2.32, 2.85)	5.48 (4.54, 6.62)
	2003–2007	2.62 (2.37, 2.89)	5.06 (4.25, 6.03)
UK	1978–1982	1.57 (1.44, 1.70)	3.73 (3.36, 4.14)
	1983–1987	2.15 (2.03, 2.27)	4.37 (4.04, 4.73)
	1988–1992	2.50 (2.39, 2.62)	4.28 (4.02, 4.57)
	1993–1997	3.85 (3.74, 3.96)	4.55 (4.36, 4.75)
	1998–2002	4.72 (4.60, 4.83)	4.77 (4.59, 4.96)
	2003–2007	5.43 (5.31, 5.55)	4.91 (4.74, 5.09)
Ireland	1988–1992	1.48 (0.71, 2.24)	4.06 (2.02, 8.14)
	1993–1997	2.91 (2.43, 3.40)	3.90 (3.09, 4.92)
	1998–2002	3.61 (3.18, 4.05)	4.88 (4.00, 5.95)
	2003–2007	4.22 (3.77, 4.67)	4.70 (3.95, 5.58)
Netherlands	1988–1992	1.93 (1.75, 2.11)	3.87 (3.41, 4.39)
	1993–1997	2.60 (2.42, 2.77)	4.49 (4.05, 4.97)
	1998–2002	3.85 (3.65, 4.05)	4.99 (4.57, 5.44)
	2003–2007	5.32 (5.10, 5.54)	5.70 (5.28, 6.15)
France	1983–1987	1.04 (0.82, 1.26)	8.85 (5.49, 14.26)
	1988–1992	1.05 (0.87, 1.23)	6.75 (4.81, 9.47)
	1993–1997	1.32 (1.14, 1.50)	7.00 (5.30, 9.25)
	1998–2002	1.88 (1.70, 2.06)	10.31 (8.13, 13.07)
	2003–2007	2.05 (1.87, 2.23)	9.15 (7.41, 11.30)
Czech	1988–1992	0.65 (0.55, 0.76)	8.11 (5.74, 11.47)
	1993–1997	0.90 (0.79, 1.02)	10.84 (7.73, 15.2)
	1998–2002	1.16 (1.03, 1.29)	9.79 (7.42, 12.92)
	2003–2007	1.65 (1.50, 1.80)	9.27 (7.42, 11.58)
Slovak	1988–1992	0.48 (0.34, 0.62)	6.50 (3.66, 11.54)
	1993–1997	0.61 (0.46, 0.76)	8.66 (5.04, 14.87)
	1998–2002	0.69 (0.52, 0.85)	5.27 (3.54, 7.86)
	2003–2007	0.73 (0.57, 0.88)	8.18 (5.14, 13.01)
Switzerland	1988–1992	0.94 (0.73, 1.15)	4.83 (3.40, 6.87)
	1993–1997	1.36 (1.10, 1.62)	4.80 (3.55, 6.48)
	1998–2002	2.41 (2.05, 2.77)	8.56 (6.06, 12.10)
	2003–2007	2.20 (1.94, 2.46)	6.2 (4.93, 7.78)
Spain	1988–1992	0.56 (0.45, 0.67)	6.82 (4.49, 10.34)
	1993–1997	0.65 (0.51, 0.78)	6.44 (4.30, 9.64)

	1998–2002	1.07 (0.94, 1.19)	8.93 (6.61, 12.05)
	2003–2007	1.37 (1.24, 1.50)	9.05 (7.09, 11.55)
Italy	1983–1987	0.42 (0.27, 0.57)	6.92 (3.37, 14.18)
	1988–1992	0.58 (0.48, 0.69)	8.20 (5.34, 12.59)
	1993–1997	0.60 (0.52, 0.69)	6.85 (5.15, 9.11)
	1998–2002	0.74 (0.66, 0.82)	6.39 (5.17, 7.91)
	2003–2007	0.75 (0.69, 0.82)	7.58 (6.28, 9.15)
India	1988–1992	0.45 (0.34, 0.56)	4.10 (2.83, 5.93)
	1993–1997	0.29 (0.22, 0.36)	2.56 (2.00, 3.26)
	1998–2002	0.30 (0.22, 0.37)	2.57 (2.00, 3.30)
	2003–2007	0.24 (0.19, 0.30)	2.79 (2.19, 3.55)
Japan	1978–1982	0.18 (0.11, 0.24)	3.38 (2.12, 5.37)
	1983–1987	0.22 (0.15, 0.30)	4.06 (2.48, 6.63)
	1988–1992	0.26 (0.21, 0.32)	6.89 (4.46, 10.62)
	1993–1997	0.37 (0.31, 0.42)	8.05 (5.72, 11.35)
	1998–2002	0.33 (0.28, 0.39)	7.60 (5.48, 10.54)
	2003–2007	0.37 (0.32, 0.42)	7.17 (5.50, 9.35)
Hong Kong	1988–1992	0.81 (0.58, 1.03)	2.75 (2.08, 3.62)
	1993–1997	0.62 (0.45, 0.79)	3.13 (2.28, 4.31)
	1998–2002	0.39 (0.28, 0.51)	4.13 (2.70, 6.32)
	2003–2007	0.21 (0.13, 0.29)	3.10 (1.98, 4.84)

CI: confidence interval; RD: risk difference; RR: relative risk measured by the male-to-female ratio in the age-standardized incidence rate using the World Health Organization (WHO) World Standard Population 2000 as the reference; UK: the United Kingdom; USA: the United States of America.

[a]In 1/100,000 person-years

[b]Surveillance, Epidemiology, and End Results Program (9 registries).
